# A Manual of Organic Materia Medica

**Published:** 1890-05

**Authors:** 


**A Manual of Organic Materia Medica**, being a guide to Materia Medica of the Vegetable and Animal Kingdoms, for the use of students, druggists, pharmacists, and physicians. By JOHN M. MAISCH, Ph. M., Phar. D., Professor of Materia Medica and Botany in the Philadelphia College of Pharmacy. Fourth edition, with 259 illustrations. Philadelphia: Lea Brothers & Co. 1890.

The scope of this manual embraces the drugs of animal and vegetable origin recognized by the pharmacopeias of the United States and Great Britain, supplemented by important non-officinal drugs, and by others recently introduced or revived, which seems to deserve attention. In view of the approaching revision of the United States Pharmacopeia, the drugs have been arranged according to origin, in which Benthani's and Hooker's Geneva Plantarum has been followed.

The drugs first discovered are of animal origin, then cellular vegetable drugs, and lastly, drugs without cellular stricture. Each drug is carefully described, giving its origin, habitat, description, constituents, properties, dose, and when necessary, its antidote. The illustrations serve to make the text more lucid and interesting, and conveys to the physician some idea of the agent he so often employs. An alphabetical classification, and the classification according to origin, are given at the end of the work. The illustrations are numerous and nicely executed, adding to the excellent typographical work done by this house.

